# Immune mechanisms associated with sex-based differences in severe COVID-19 clinical outcomes

**DOI:** 10.1186/s13293-022-00417-3

**Published:** 2022-03-04

**Authors:** Cosby G. Arnold, Anne Libby, Alexis Vest, Andrew Hopkinson, Andrew A. Monte

**Affiliations:** Department of Emergency Medicine, University of Colorado-Anschutz Medical Campus, 12401 East 17th Avenue, 7th Floor, Aurora, CO 80045 USA

**Keywords:** Sex, Gender, SARS-CoV-2, COVID-19

## Abstract

**Background:**

Although biological males and females are equally likely to become infected with severe acute respiratory syndrome coronavirus 2 (SARS-CoV-2), evidence has mounted that males experience higher severity and fatality compared to females.

**Main:**

The objective of this review is to examine the existing literature on biological mechanisms underlying sex-based differences that could contribute to SARS-CoV-2 infection clinical outcomes. Sex-based differences in immunologic response and hormonal expression help explain the differences in coronavirus disease 2019 (COVID-19) outcomes observed in biological males and females. X inactivation facilitates a robust immune response to COVID-19 in females, who demonstrate a more profound antibody response and faster recovery when compared to males. Low testosterone levels also help explain the dysregulated inflammatory response and poor outcomes observed in some males with COVID-19. Gender differences in health expression and behaviors further compound these observed differences.

**Conclusion:**

Understanding the biology of sex-based differences in COVID-19 severity and mortality could help inform preventative measures, treatment decisions, and development of personalized, sex-specific therapies.

## Highlights

- Although most COVID-19 patients develop only mild disease, some mount a pronounced inflammatory response that results in severe pneumonia, respiratory failure, and death.
- Male sex is a known risk factor for severe outcomes in COVID-19.
- Biological sex-based differences in COVID-19 severity and mortality are influenced by genetic and hormonal differences at the cellular and immunologic level.
- Biological sex-based differences are further complicated by environmental factors, namely social and cultural norms, that define gender roles which impact risk of infection and severe outcomes.
- Understanding sex-based differences in COVID-19 outcomes will help inform clinical decision algorithms and treatment strategies to optimize the care of COVID-19 patients.

## Background

Coronavirus disease 2019 (COVID-19), caused by the novel severe acute respiratory syndrome coronavirus 2 (SARS-CoV-2), emerged in December 2019 and quickly overwhelmed healthcare systems across the globe. The World Health Organization (WHO) declared COVID-19 as a public health emergency on January 30, 2020 and a pandemic on March 11, 2020. Although most COVID-19 patients develop only mild symptoms, some develop severe pneumonia, acute respiratory distress syndrome (ARDS), and multi-organ failure; as of February 2022, there were nearly 400 million reported cases and 6 million deaths worldwide [1, 2, 3].

Emerging epidemiologic studies have identified compelling and consistent sex differences in COVID-19 outcomes. The COVID-19 Sex-Disaggregated Data Tracker, developed by the Global Health 50/50 research initiative, showed similar numbers of COVID-19 cases in people identifying as men and women. However, men were more likely to develop severe illness and have higher case fatality rates, especially when older than 60 years [4]. In a meta-analysis of 3,111,714 infected cases from 46 countries and 44 US states, male patients had higher odds of ICU admission (odds ratio (OR) = 2.84; 95% confidence interval (CI) = 2.06, 3.92) and death (OR = 1.39; 95% CI = 1.31, 1.47) compared to female patients [5]. An analysis of 308,010 patients hospitalized with COVID-19 at US academic centers found that, compared to females, males had a higher mortality rate (13.8% vs. 10.2%, *p* < 0.001) across all age groups, race/ethnicity, payers, and preexisting comorbidities [6]. These findings are consistent with prior, smaller coronavirus outbreaks such as SARS-CoV in 2002, and middle east respiratory syndrome (MERS-CoV) in 2012. In both these outbreaks males were found to be at higher risk for poor clinical outcomes compared to females [7–10, 11]. In this paper we examine how biological sex in COVID-19 infection could help inform approaches to risk stratification and treatment, and explore possible sociodemographic confounders such as ancestry, gender and age. [10, 12, 13].

## Main text

### Sex as a biological variable: immune response

Sex-based differences in COVID-19 outcomes are mediated by immune response. Females mount a stronger immune responses to a variety of infections, likely due to the influence of genetic and hormonal differences on the immune system [14]. Although females tend to experience less severe disease in response to viral infection, these infections are thought to contribute to higher rates of autoimmune disease observed in this population [15]. The X chromosome encodes several genes involved in innate and adaptive immune function. These genes include pattern recognition receptors (PRR) such as toll-like receptor (TLR) 7 and TLR8 and interleukin 1 receptor associated kinase (IRAK) 1 [16]. Since biological females have two copies of these immune genes, there is both redundancy and heterogeneity in their phenotypes [17]. Because males have only a single X chromosome, they have 5% fewer heterozygous loci than females and it is hypothesized that a lack of heterozygous loci increases the risk of viral and bacterial infection and leads to increased mortality due to infection in males [18]. Females also have more plasticity in their immune response due to cell mosaicism. X inactivation leads to expression of the maternal X chromosome in some tissues, while other tissues express the paternal X chromosome in a mosaic pattern [19, 20]. This leads to tissue specific responses, which can isolate poor outcomes to single organs rather than a generalized inflammatory response that may occur in males.

Multiple elements of the immune response to viral infection vary by sex. TLR7, an endosomal receptor expressed on dendritic and B cells, recognizes viral infections and triggers a type I interferon (IFN) response [21]. TLR7 exhibits incomplete inactivation and is transcribed on both X chromosomes [22]. Higher levels of TLR7 expression in females strengthens the immune response and likely provides an advantage in response to viral infections, including COVID-19 [20, 23, 24]. A preliminary case study of young male patients with COVID-19 identified loss-of-function variants of TLR7 that were associated with severe clinical disease [24]. Robust T cell activation and IFN response following TLR7 activation in females has also been observed and likely contributes to better prognosis [20]. Although T cell lymphopenia has been observed in both sexes with COVID-19, females mount a stronger T cell response, particularly with CD8 + T cells [25]. A poor T cell response is also correlated with age and associated with worse outcomes in male patients [25]. In patients with severe disease, the protective SARS-CoV-2 immunoglobulin G (IgG) antibody levels are higher in female patients compared to male patients and may contribute to improved clinical outcomes [26]. SARS-CoV-2 IgG levels more quickly rise and peak in female versus male patients, consistent with a sluggish antibody response and prolonged recovery from infection in males [27]. Higher IgG titers are also observed in the convalescent plasma of male donors, possibly reflective of more severe disease resulting in more antibody production [28, 29]. Interestingly, seroconversion status is associated with distinct underlying pathophysiology in COVID-19, with low antibody titers associated with inflammatory biomarkers, although these findings were not stratified by sex. [30].

Sex hormones play an important role in immune function and response to viral infections. Differences in sex hormone levels help explain the immune response and COVID-19 severity in males compared to females. In a single-center cohort study of patients with COVID-19, lower testosterone levels and increased estradiol to testosterone ratio during hospitalization were associated with higher inflammatory cytokine concentrations, increased disease severity, ventilator use, ICU admission, and mortality [31]. This difference persisted independent of other risk factors for severe outcomes, including age, BMI, medical comorbidities, smoking, and race. [31].

Testosterone has suppressive effects on immune function, and estrogen may have either positive or negative effects, depending on the level [32]. For males, testosterone levels remain stable up to 70 years of age; however, in females estrogen levels fluctuate during the menstrual cycle and drop after menopause. Estrogen levels, whether high or low, have varying effects on immune response. Higher estrogen levels inhibit the pro-inflammatory innate immune response, enhance T helper 2 and humoral immune response, and exert protective effects on endothelial cell function [33]. However, at low doses, estrogen promotes pro-inflammatory responses, including increased production of pro-inflammatory cytokines and enhanced cell-mediated immune response. Low estrogen levels induce monocyte differentiation into inflammatory dendritic cells, increase production of IL-4 and IFNα, and promote T helper 1 and cell-mediated immune response [20, 33, 34]. These immune modifications across the life course help dictate the strength of the inflammatory response and influence the likelihood of progression to severe clinical outcomes for patients with COVID-19, with numerous documented sex differences. No data on differences in outcomes based upon timing of infection in relation to the menstrual cycle have yet been published.

Low testosterone levels correlate with COVID-19 severity in males [35, 36]. Testosterone suppresses the production of pro-inflammatory cytokines and facilitates the differentiation of regulatory T cells that act to suppress the immune response. While clearly not the only covariate, males with testosterone insufficiency, as observed in elderly and comorbid patients, are predisposed to a higher systemic inflammatory response [37, 38]. Low testosterone levels are associated with increased all-cause mortality due to cardiovascular events and may increase cardiovascular risk in males with COVID-19. Testosterone is critical to platelet and coagulative homeostasis; males with low testosterone levels may be predisposed to thromboembolic events in COVID-19 [39]. Testosterone deficiency may also increase angiotensin converting enzyme 2 (ACE2) receptor expression, thereby facilitating SARS-CoV-2 entry into host cells increasing lung damage and respiratory failure [20, 39, 40]. These effects are more pronounced in obese males, since ACE2 receptors are expressed in adipose tissue which facilitates an even greater systemic response. [41].

## Confounders and influences on observed sex-based disparities in COVID-19 outcomes

In addition to biological sex-based differences, gender, as defined by social and cultural norms, plays a role in risk for infection and progression to severe outcomes. Gender differences are commonly described for groups identifying as women and men, leaving unmeasured variation in transgender or individuals of other gender identities, who may not be identifiable in existing studies, thereby confounding all gender-specific measures. In the United States, approximately 0.4% of the population identify as transgender [42]. In terms of co-occurring disorders, women are documented as having experienced higher levels of stress, anxiety, and depression as a result of the pandemic compared to men [43]. Gender influences occupation, with more than 75% of the U.S. healthcare workforce identifying as women, and women are more likely to be “essential workers.” [44] Women are more likely to seek medical care and more likely to follow public health guidance regarding hand-washing and mask-wearing [44]. Smoking is more common in men and thought to increase risk for severe disease and death, potentially due to underlying lung disease and through increased expression of ACE2 and modulation of pro-inflammatory cytokines [45]. As these examples suggest, gender-informed behaviors may increase risk for acquisition of infection and severe clinical outcomes. Race/ethnicity is a demonstrated indicator in severity [46], with an unexplained role of structural racism [47]. Older age is also associated with worse outcomes, as are interactions among these key variables [46]. For example, Black and Hispanic men age 65 years and older tend to have more severe progression, more need for hospitalization, and a higher likelihood of death.^48^ These clinical outcomes potentially reflect social and cultural norms rooted in unmeasured gender differences.

## Future research needs

Additional studies are needed to understand the impact that genetics, hormones, and innate and adaptive immune functional differences play in COVID-19 outcomes. Identifying hormone levels and biomarkers associated with sex-based differences in clinical outcomes may allow for earlier prediction of anticipated clinical course and inform therapeutics. This approach will likely have applications beyond COVID-19, and facilitate understanding of response to other viral infections in preparation for the next pandemic.

## Conclusions

Sex-based differences in COVID-19 outcomes are multifactorial. Understanding the mechanisms underlying these differences could help predict patient outcomes and inform clinical decision-making to facilitate early treatment and disposition decisions. Recognition of sex-based differences can also help inform future research efforts to develop targeted, sex-specific therapies in COVID-19.

## Data Availability

Not applicable.

## Abbreviations

PASC: Post-acute sequelae of SARS-CoV-2
COVID-19: Coronavirus disease 2019
SARS-CoV-2: Severe acute respiratory syndrome coronavirus 2
WHO: World Health Organization
ARDS: Acute respiratory distress syndrome
ICU: Intensive care unit
MERS-CoV: Middle east respiratory syndrome
TLR7: Toll-like receptor 7
IFN: Interferon
IgG: Immunoglobulin G
ACE2: Angiotensin converting enzyme 2
